# Portal vein thrombosis following laparoscopic cholecystectomy complicated by dengue viral infection: a case report

**DOI:** 10.1186/1752-1947-5-126

**Published:** 2011-03-30

**Authors:** Dilip Dan, Kevin King, Shiva Seetahal, Vijay Naraynsingh, Seetharaman Hariharan

**Affiliations:** 1 Department of Clinical Surgical Sciences, University of the West Indies, St Augustine, Trinidad and Tobago

## Abstract

**Introduction:**

Portal vein thrombosis is an uncommon post-operative complication following abdominal surgery. Although therapeutic anticoagulation is recommended, this treatment may be questionable when the patient has an associated bleeding diathesis.

**Case presentation:**

We report a case of a 63-year-old woman of Asian Indian ethnicity who developed portal vein thrombosis following an uneventful laparoscopic cholecystectomy for symptomatic gallstones. Her condition was further complicated by dengue viral infection in the post-operative period, with thrombocytopenia immediately preceding the diagnosis of portal vein thrombosis. The etiological connections between dengue viral infection with thrombocytopenia, laparoscopic cholecystectomy, portal vein thrombosis as well as the treatment dilemmas posed in treating a patient with portal vein thrombosis with a bleeding diathesis are discussed.

**Conclusion:**

When portal vein thrombosis occurs in patients with contraindications to anticoagulation, there is a role for initial conservative management without aggressive anticoagulation therapy and such patients must be approached on an individualized basis.

## Introduction

Portal vein thrombosis (PVT) is one of the recognized complications in the post-operative period following abdominal surgeries, although it is uncommonly reported in the literature. PVT may usually manifest in a patient who is in a hypercoagulable state, but to the best of our knowledge, has never been reported in a patient with thrombocytopenic hemorrhagic disorder. We report a patient who presented with PVT, five days after an uneventful laparoscopic cholecystectomy. She was simultaneously diagnosed with thrombocytopenia secondary to dengue virus infection. This case is noteworthy in that it represents an unusual constellation of diseases and poses interesting challenges regarding the seemingly contradictory fundamentals of management.

## Case presentation

A 63-year-old woman of Asian Indian ethnicity presented with complaints of biliary colic, which was worsening over a period of six months. She denied jaundice, fevers or weight loss. She had a past medical history of hypertension, diabetes mellitus and ischemic heart disease; she had received coronary angioplasty and stenting two years prior to the presentation. She was on 81 mg of aspirin and 75 mg of clopidogrel daily. Significantly, she had no previous hormone use and no history of deep vein thrombosis. On clinical examination, our patient was afebrile, anicteric, had normal body habitus and a benign abdomen; examination of other systems was largely unremarkable.

Ultrasound examination of her abdomen confirmed the clinical suspicion of cholelithiasis, however, there was no evidence of acute inflammation and her bile ducts appeared normal. All associated structures, including her liver and portal vein, were found to be normal. Her laboratory investigations included complete blood count, renal function tests, liver function tests (LFT) and coagulation profile, which were all within normal limits.

She was scheduled for an elective laparoscopic cholecystectomy (LC) two weeks later. Her pre-operative instructions included cessation of anti-platelet drugs five days before surgery.

Our patient underwent an uneventful procedure which lasted for 25 minutes. Her gallbladder was categorized as Class I and the insufflation pressures of pneumoperitoneum was never higher than 12 mmHg intra-operatively. She was discharged on the first post-operative day with instructions that included restarting her aspirin and clopidogrel on the same day.

**Figure 1:**
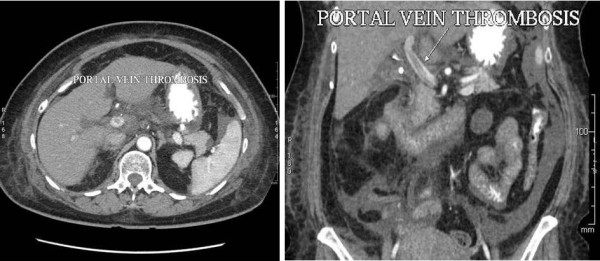
**CT Abdomen showing thrombus in the portal vein (coronal and sagittal views)**.

Our patient returned to the hospital on the fourth post-operative day with intractable nausea, vomiting and diarrhea. Notably, fever was absent. She had mild-to-moderate dehydration and was admitted for rehydration therapy and further investigation. Laboratory reports showed elevation of liver enzymes and a platelet count of 16,000/μL. She was hemodynamically stable and showed no other signs of sepsis syndrome. There were no symptoms or signs of upper gastro-intestinal bleeding and hence no endoscopy was undertaken. An ultrasound of her abdomen revealed normal bile ducts and ascites. A computed tomography (CT) scan with dual contrast confirmed the presence of minimal ascites and additionally demonstrated a thrombus in the portal vein Figure 1.

With the diagnosis of PVT, anticoagulation was contemplated but withheld owing to her thrombocytopenia. Dengue is endemic to Trinidad, and so on admission - based on her clinical presentation and a high index of suspicion - dengue viral antibody titers (IgG, IgM) were sought for, which returned positive. This diagnosis reinforced the decision not to anticoagulate. She was treated conservatively with intravenous fluids, antibiotics and careful observation, which included daily laboratory investigations. With this treatment regimen, she showed gradual improvement of her clinical symptoms as well as her laboratory values. LFT's normalized and her platelet count started to improve. The trends of the various hematological and biochemical parameters during her hospital stay are shown in Table 1.

**Table 1:** Blood counts and liver enzymes during hospital stay

Parameter	Day-1	Day-2	Day-3	Day-4	Day-5	Day-6	Day-7
**Hb **(g/dL)	11.9	11.4	11.1	11.4	10.9	11.3	11.4
**WBC **(× 10^3^/μL)	6.6	6.0	5.1	4.9	4.6	5.0	5.1
**Platelet Counts** (130,000-140,000/μL)	16,000	27,000	30,000	64,000	112,000	168,000	242,000
**AST** (0-40 units)	188	146	96	-	-	-	36
**ALT** (0-38 units)	64	44	35	-	-	-	30
**GGT** (4-471 units)	-	119	91	-	-	-	90
**Bilirubin** (< 1.1 mg/dL)	-	0.4	0.5	-	-	-	0.4
**ALP** (30-306 units)	-	788	708	-	-	-	203
**INR**	-	1.0	-	-	-	1.93	2.03

Hb: Hemoglobin

WBC: White Blood Cell count

AST: Aspartate transaminase

ALT: Alanine transaminase

GGT: Gamma-glutamyl transferase

ALP: Alkaline Phosphatase

INR: International Normalized Ratio

Values in brackets are laboratory normal ranges.

On the 10^th ^post-operative day, six days after her diagnoses of PVT and dengue viral infection, her symptoms completely ameliorated and her platelet count had risen to 125,000/μL. She did not have any complications of PVT such as variceal bleeding. At this time, 40 mg per day of enoxaparin was started. Repeat ultrasound done at this time revealed resolution of the PVT. She was discharged on day 14 after being placed on 5 mg of warfarin daily for three months. She is currently off the anticoagulation; aspirin and clopidogrel have been restarted and she has no residual clinical sequelae pertaining to PVT and/or dengue infection.

## Discussion

Balfour and Stewart reported a case of PVT as early as 1868. With the advent and easy availability of ultrasonography, PVT is being diagnosed much more frequently than before. In a Swedish study published during 2005, autopsies of over 23,000 hospitalized patients were reviewed and the incidence of PVT (representing the risk to the general population) was found to be 1% [1].

Hypercoagulable state is a major risk factor for developing PVT. This encompasses a spectrum that includes inherent conditions such as Factor V Leiden or Protein C deficiency, as well as acquired predilections such as neoplastic disease. Soogard *et al. *have described the risk factors for PVT of which prothrombotic disorders were the predominant risk factors amounting to 28%. Other major risk factors reported in this study were abdominal inflammation (19%), cirrhosis of liver (13%), malignancies (11%), abdominal intervention (8%), abdominal infection (8%) or idiopathic (13%) [2]. This spectrum includes gall stones and cholecystectomy (abdominal inflammation and intervention), which were the main risk factors in our patient. Her coagulation studies were normal pre-operatively and she had no risk factors for thrombophilia such as hormone use or previous history of thrombotic episodes, neoplastic disease or cirrhosis of the liver. This may point to the possibility that our patient would have developed PVT following LC, which is one of the rare causes of PVT, and has been reported previously [3-5].

PVT following LC is rare in itself; however, the clinical scenario in our situation was further confounded by the dengue virus infection. Dengue virus belongs to the genus *Flavivirus *and is endemic to many countries in the Caribbean. There are four known serotypes and two distinct clinical syndromes - Dengue fever and Dengue Hemorrhagic Fever (DHF). The former is characterized by the classic features of fever, myalgia, arthralgias, headache and petechial rash. Thrombocytopenia and leucopenia are present but usually mild. Symptoms such as nausea, vomiting and diarrhea are less common. DHF causes a more severe thrombocytopenia and leucopenia, with plasma leakage from capillaries [6]. When our patient presented to us following LC on the fourth postoperative day, she had been clinically dehydrated secondary to the vomiting, diarrhea and possible capillary leak caused by dengue viral infection. Dehydration is a risk factor for thrombosis due to attendant hemoconcentration. We speculate that this could have been the most probable correlation between the dengue viral infection and PVT in our patient.

However, there may be alternate and/or additional relationships between dengue fever and PVT. Animal studies have shown that there is a cross-talk between dengue fever and thrombotic processes [7]. Dengue viral infection may be responsible for the down regulation of thrombomodulin-thrombin-protein C complex formation reducing activated protein C, activating the link between coagulation-inflammation pathways [8]. Dengue virus activates endothelial cells, alters the parameters of hemostasis and increases the expression of thrombomodulin [9,10]. Furthermore, an autoimmune theory has been suggested for the pathophysiology of the symptoms following dengue viral infections. Lin *et al. *described host antibodies formed against non-structural protein in the dengue virus that had cross-reactivity with endothelial cells in the host, which can lead to inflammatory responses [11]. Theoretically this pathogenesis may predispose to thrombus formation, although at this time there is no clear evidence or published data to support this extrapolation. Finally, cardiolipin antibodies are of the IgG type and are known to predispose to PVT. Krnic-Barrie *et al. *have suggested the possibility of other classes of IgG having similar thrombophilic properties [12]. Dengue virus has the ability to induce IgG, but this is at a later stage of infection or at re-exposure. Although this theory may seem implausible, the possibility cannot be completely ruled out.

Apart from establishing the diagnostic relationships between PVT and dengue viral infection, the dilemmas posed during treatment are perhaps more relevant to the practicing clinician. In acute PVT setting, the sooner the institution of anticoagulants, the better will be the outcome of patients [13]. However, the predicament of having to treat a blood clot in a patient who is at risk for excessive bleeding is the conundrum, although it is not an uncommon occurrence. Usually, the more life-or-limb threatening condition is addressed aggressively, while careful observation and monitoring would be employed on its apparent nemesis. We adopted a similar strategy for our patient. PVT can complicate with gastropathy, ascites and, most dangerously, with gastro-esophageal varices and possible hemorrhage. Dengue infection can cause severe thrombocytopenia, vascular leakage and life-threatening hemorrhage. There is evidence that Dengue Virus-induced tissue plasminogen activator regulated by interleukin-6 may be responsible for the bleeding in DHF [14]. Often the clinical course is unpredictable and Dengue Hemorrhagic Shock Syndrome is an extremely lethal entity [6]. In our patient, the PVT was largely asymptomatic. In fact if she had not suffered the dengue infection in the post-operative period, PVT might not have been diagnosed at all. Sub-clinical PVT is not uncommon. Since our patient was asymptomatic with respect to her PVT, she was managed initially without anticoagulation. A similar approach has been adopted by other authors [1,15]. Following the resolution of her viral infection she was placed on anticoagulation because the risk of hemorrhage was significantly reduced at this time.

## Conclusion

PVT is a post-operative complication following laparoscopic procedures and is being diagnosed much more frequently than before due to advances in imaging techniques. In the setting of the tropics, dehydrating viral illnesses may also precipitate PVT. When PVT occurs in patients with contraindications to anticoagulation, there is a role for initial conservative management without aggressive anticoagulation therapy and such patients must be approached on an individual basis.

## Consent

Written informed consent was obtained from the patient for publication of this case report and any accompanying images. A copy of the written consent is available for review by the Editor-in-Chief of this journal.

## Competing interests

The authors declare that they have no competing interests.

## Authors' contributions

DD, KK and SS clinically managed the patient and initially drafted the manuscript. VN and SH interpreted the patient data and were major contributors in writing and revising the manuscript. All authors read and approved the final manuscript.
